# Titration-based normalization of antibody amount improves consistency of ChIP-seq experiments

**DOI:** 10.1186/s12864-023-09253-0

**Published:** 2023-04-04

**Authors:** Ariel Caride, Jin Sung Jang, Geng-Xian Shi, Sam Lenz, Jian Zhong, Kwan Hyun Kim, Mariet Allen, Keith D. Robertson, Gianrico Farrugia, Tamas Ordog, Nilüfer Ertekin-Taner, Jeong-Heon Lee

**Affiliations:** 1 Epigenomics Development Laboratory, Epigenomics Program, Center for Individualized Medicine, Mayo Clinic, Stabile Building 12-04, 200 First Street SW, Rochester, MN USA; 2 Medical Genome Facility, Center for Individualized Medicine, Mayo Clinic, Rochester, MN USA; 3 Department of Neuroscience, Mayo Clinic, Jacksonville, FL USA; 4 Department of Molecular Pharmacology and Experimental Therapeutics, Mayo Clinic, Rochester, MN USA; 5 Enteric Neuroscience Program, Mayo Clinic, Rochester, MN USA; 6 Department of Physiology and Biomedical Engineering, Mayo Clinic, Rochester, MN USA; 7 Division of Gastroenterology and Hepatology, Department of Medicine, Mayo Clinic, Rochester, MN USA; 8 Department of Neurology, Mayo Clinic, Jacksonville, FL USA; 9 Department of Biochemistry and Molecular Biology, Mayo Clinic, Rochester, MN USA; 10 Division of Experimental Pathology and Laboratory Medicine, Department of Laboratory Medicine and Pathology, Mayo Clinic, Rochester, MN USA

**Keywords:** Chromatin quantification, Chromatin immunoprecipitation, Antibody titer, H3K27ac, Experimental consistency

## Abstract

Chromatin immunoprecipitation (ChIP) is an antibody-based approach that is frequently utilized in chromatin biology and epigenetics. The challenge in experimental variability by unpredictable nature of usable input amounts from samples and undefined antibody titer in ChIP reaction still remains to be addressed. Here, we introduce a simple and quick method to quantify chromatin inputs and demonstrate its utility for normalizing antibody amounts to the optimal titer in individual ChIP reactions. For a proof of concept, we utilized ChIP-seq validated antibodies against the key enhancer mark, acetylation of histone H3 on lysine 27 (H3K27ac), in the experiments. The results indicate that the titration-based normalization of antibody amounts improves assay outcomes including the consistency among samples both within and across experiments for a broad range of input amounts.

**Supplementary Information:**

The online version contains supplementary material available at 10.1186/s12864-023-09253-0.

## Background

Chromatin immunoprecipitation (ChIP) assay combined with quantitative PCR or next-generation sequencing is a frequently used and critical methodology to directly analyze the binding sites of chromatin-associated proteins or the locations of histone modification locally or genome-wide [1–8]. It is well known that the success of a ChIP experiment is governed by the specificity of the antibody and the degree of enrichment achieved in the immunoprecipitation stage. Thus, the Encyclopedia of DNA Elements (ENCODE) Consortium provided the necessary requirements of antibody for immunoprecipitation specificity and enrichment, emphasizing ChIP-validated antibodies must be used in targeted and genome-wide ChIP applications [9, 10]. However, the fundamental aspect of immunoprecipitation regarding antibody titer has not been addressed well. We hypothesize the antibody titer in ChIP reaction is critical for experimental outcome and consistency among samples.

Chromatin input is typically generated by fragmenting bulk chromatin into sizes of mono- to tri-nucleosomes using sonication, micrococcal nuclease (MNase), or the combined approaches in ChIP applications [7, 8]. However, the yield of soluble chromatin (i.e., chromatin input in ChIP experiment) is likely dependent on the experimental conditions such as sample type, fixation, fragmentation method, and sample preservation [11–14]. It is more challenging to estimate the amount of soluble chromatin for solid tissue samples. The cellularity (i.e., the number of nucleated cells) of solid tissue is often not available and is variable in samples even from the same tissue type. Thus, the amount of chromatin input available to a given ChIP reaction is expected to be highly variable and unpredictable.

The antibody titer of each antibody is experimentally determined to identify the amount of antibody related to antigen yielding the optimal signal-to-noise ratio in immunoprecipitation or other experiments [15, 16]. For protein extract-based immunoprecipitation experiments, a titration experiment is typically performed with multiple antibody concentrations ranging from 1 to 10 μg for ~ 5,000 μg of protein extract. In general, too much antibody over the optimal titer increases background noise and too little antibody yields less target of interest [17, 18]. However, antibody titration in the context of ChIP experiment was inadequately documented. Furthermore, it has been a challenge to use the optimal titer of antibody due to the lack of a quick and reliable quantification method for chromatin input. Typically, the amount of chromatin input is indirectly determined as DNA amount after purification, and it takes several hours to days and includes multiple steps such as cross-linking reversal, treatment of RNase and proteinase K, DNA purification, and DNA quantification [19]. Currently, no methods are available to quantify chromatin amount quickly and reliably in the context of ChIP applications. We believe quantification of chromatin input amount immediately after preparation is highly beneficial and enables the researcher to normalize the antibody amount to the optimal titer, ensuring the ChIP reaction condition is consistent between samples or throughout multiple independent experiments.

In this study, we validated a quick and direct DNA-based measurement of soluble chromatin that provides reliable and quantitative measures of chromatin input that is highly comparable with the amount of chromatin determined by purified DNA. This approach permits the accurate quantification of the available amount of chromatin input in a broad range from individual samples, and allows the optimal titer of antibody to be employed in downstream ChIP reactions. The results indicate that normalizing antibody amount to the optimal titer improves the overall outcome of data quality with high experimental consistency in a single experiment or recurring experiments over time.

## Results

### A quick and easy method to quantify ChIP input from individual chromatin samples

**Fig. 1:**
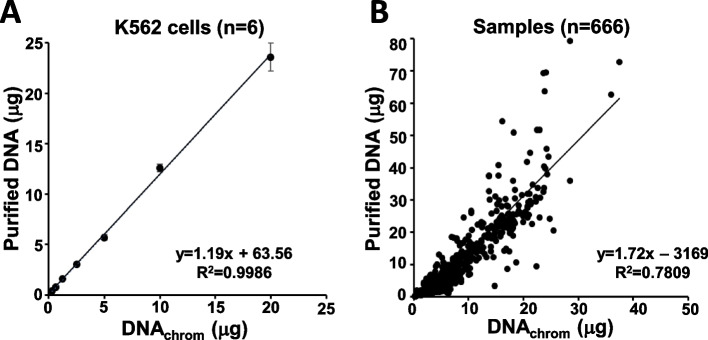
Direct measurement of DNA content in chromatin input enables to quantify the amounts of usable input from individual samples. **A** The DNA content directly measured in chromatin input (DNA_chrom_) is correlated with the amount of purified DNA. Chromatin input was prepared from fixed K562 cells. DNA amount was directly measured in chromatin input ranging 0.3 – 20 µg by the Qubit dsDNA high sensitivity assay and compared with the amount of purified DNA. The Qubit assay was performed by incubating 2 µl of chromatin input with 198 µl of Qubit reagent. The data was presented as mean ± SD from 2 experiments performed in triplicates. Coefficient of determination (R^2^) was calculated by the linear regression model. **B** Correlation between DNA_chrom_ and the amount of chromatin input determined by purified DNA in various sample types. Chromatin input was prepared from cell lines (*n* = 78), solid tissues (*n* = 534), and samples derived from peripheral blood (*n* = 54). DNA_chrom_ was measured from 0.5—1% of chromatin input in individual ChIP reactions and compared with the amount of chromatin input determined by purified DNA

First, we investigated whether quantifying DNA directly in freshly prepared, individual chromatin samples would accurately reflect their DNA content and could therefore serve as a basis for determining the amount of solubilized chromatin input for ChIP reactions. Chromatin input was prepared from 30 million fixed K562 cells as previously described [19]. DNA content of the chromatin input (defined as DNA_chrom_) was directly measured from 0.2% of total input by the Qubit assay [20, 21], a high-sensitivity method specific to double-stranded (ds) DNA, following the manufacturer’s instructions. DNA was then purified from 1% of the total input containing 0.3 to 20 µg of DNA_chrom_ after cross-linking reversal, RNase A and proteinase K treatments, and column purification and quantified by the Qubit assay. The amount of DNA purified from the chromatin input showed strong linear correlation (R^2^ = 0.99) with DNA_chrom_ (Fig. 1A). Next, we confirmed the linear correlation between the amounts of purified DNA and DNA_chrom_ in 666 different chromatin inputs prepared from various sample types in the context of ChIP experiments (Additional file 1: Table S1). DNA_chrom_ was measured from less than 1% of chromatin input and the measurements typically took less than 5 min to complete. Remarkably, we detected robust linear correlation across all samples and a wide range of purified DNA amounts (0.19 µg to 76.78 µg; R^2^ = 0.74) (Fig. 1B) despite a variability in the slope of the linear regression obtained in the 6 different sample types analyzed (1.08–2.60; Additional file 2: Fig. S1). Interestingly, we obtained the highest proportional yields of purified DNA in buffy coat and PBMC samples with linear regression slopes of 1.82 and 2.60, respectively (Additional file 2: Fig. S1E and F). These observations may reflect a variability in the reactivity of Qubit reagent with chromatin input or in the efficacy of DNA isolation. Altogether, these results indicate that Qubit assay performed directly in a small fraction of the chromatin input allows quick, easy, and sensitive quantification of the chromatin input immediately after its preparation, enabling the optimization of ChIP antibody:chromatin ratios in individual samples.

### Determination of optimal antibody titer by ChIP-qPCR

**Fig. 2:**
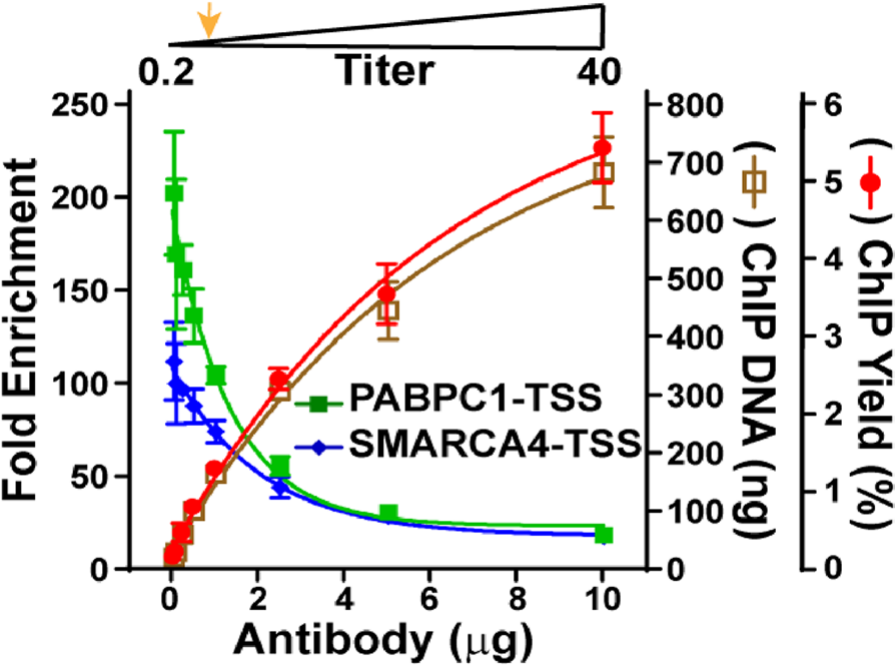
Determination of the optimal antibody titer using DNA_chrom_. Chromatin input was prepared from 40 million fixed K562 cells and DNA_chrom_ was measured. 0.05 to 10.0 µg of ChIP-validated anti-H3K27ac antibody was incubated with 10 µg of DNA_chrom_ in ChIP reactions. Fold enrichment of H3K27ac-positive *PABPC1*-TSS and *SMARCA4*-TSS loci against a negative *MYT1*-TSS locus was shown in the left y-axis to access the specificity of individual ChIP reaction. The DNA amount obtained after ChIP (ChIP DNA) and ChIP yield were shown in the right Y- axes to access the yield of immunoprecipitation in individual reaction. The optimal titer as the the ratio between antibody and chromatin amounts to yield 1 – 5 ng of ChIP DNA and 5 – 200 fold enrichment in multiple positive over negative loci was highlighted as orange arrow on the top, and the ratio between the antibody amount and DNA_chrom_ at the optimal titer is indicated as titer 1 (T1)

Next, we investigated whether DNA_chrom_ could be used as a reference for determining optimal antibody titers in ChIP applications. A ChIP-seq validated antibody against the histone mark acetylated at histone H3 lysine 27 (H3K27ac) (Abcam, Cat. ab4729), which has been utilized in numerous ChIP-seq experiments to identify active enhancers and promoters [22] (https://www.abcam.com/histone-h3-acetyl-k27-antibody-chip-grade-ab4729.html), was selected for the experiment. Chromatin input was prepared from 40 million fixed K562 cells and DNA_chrom_ was measured as described above. 10 μg of DNA_chrom_ was used in individual ChIP reactions with different amounts of antibody ranging from 0.05 to 10.0 µg. The size of the DNA purified from the chromatin input and immunoprecipitated chromatin showed remarkable consistency across the range of antibody titers (Additional file 4: Fig. S3A). ChIP yield, the DNA amount obtained after ChIP divided by the DNA amount of total chromatin input, was measured to access the yield of the immunoprecipitations from individual ChIP reactions. The fold enrichment, the % enrichment of a H3K27ac-positive genomic locus vs. local input (measured by ChIP-qPCR) divided by the enrichment of a H3K27ac-negative locus, was measured to access the specificity of individual reactions. ChIP yield gradually increased from 0.1% to 5.4% (corresponding to about 10 ng to 700 ng of DNA) with increasing amounts of antibody in the reaction (Fig. 2, y-axis on the right). It is noteworthy that 1 ng of ChIP DNA is already sufficient to generate libraries suitable for NGS [19]. In contrast, the fold enrichment of the H3K27ac-positive *PABPC1* transcription start site (TSS) locus over the H3K27ac-negative *MYT1*-TSS locus dramatically decreased from 202- to 18-fold (Fig. 2, y-axis on the left), resulting in an inverse linear correlation between ChIP yield and locus-specific enrichment (R^2^ = 0.86) (Additional file 4: Fig. S3B). We observed the degree of fold enrichment was dependent on the specific positive and negative genomic loci utilized in the ChIP-qPCR assay as exemplified by the *SMARCA4*-TSS and other loci (Fig. 2 and Additional file 4: Fig. S3C), but the relationship between fold enrichment and the antibody titer was similar across all loci tested. Based on these observations, we concluded the optimal range of antibody titer was 0.25 μg to 1 μg per 10 μg of DNA_chrom_, yielding at least 1 ng of purified ChIP DNA and a 5—200-fold enrichment in multiple positive over negative loci. We defined the ratio between the antibody amount and DNA_chrom_ at the optimal titer as “titer 1” (T1, marked as an orange arrow in the figures). It is noteworthy that antibody titer is specific for the antibody lot used in the experiment.

### Validation of the utility of titer optimization in ChIP reactions

**Fig. 3:**
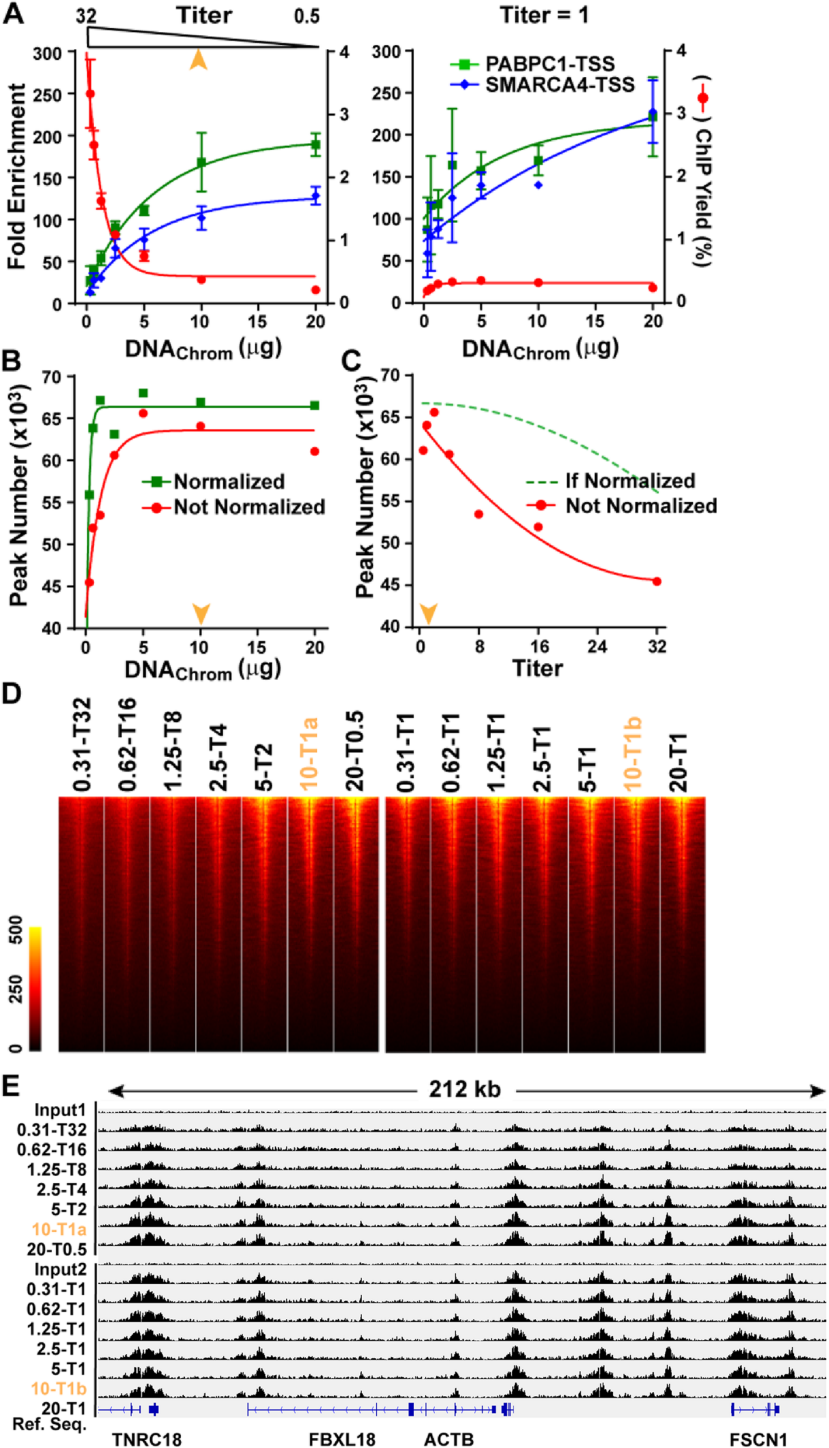
Normalized antibody amount to the optimal titer improves the experimental outcome and consistency. **A** Titration-based normalization of antibody amount to the optimal titer results in consistent ChIP yield and improved specificity. Chromatin input was prepared from fixed K562 cells. Fixed 0.25 µg of anti-H3K27ac antibody (left panel) or normalized amounts to the optimal titer (right panel) were incubated with DNA_chrom_ ranging 0.31 – 20 µg in ChIP reactions. The ratio of antibody amount/DNA_chrom_ at the optimal titer is indicated as 1 (orange arrow), and the relative antibody titers ranging 0.5 to 32 is shown on the top. The fold enrichments of positive loci over a negative locus (left Y-axis) were plotted with the amounts of chromatin input by DNA_chrom_. The % ChIP yield (right Y-axis) was calculated by DNAs isolated from chromatin input and ChIPed chromatin. The optimal titer determined in Fig. 2 was highlighted as orange arrow on the top. The experiments were performed with triplicates. **B-C** Normalization of antibody amount to the optimal titer improves peak number and consistency. The libraries were generated from the experiments described above. The peaks were called by Macs2 using FDR < 0.01. (**B**). The peak numbers obtained with fixed or normalized antibody amounts were plotted with the amounts of chromatin input by DNA_chrom_. (**C**). The peak numbers were plotted for antibody titers in the reactions with the fixed amount of antibody. The dotted line indicates the peak numbers after the normalization of antibody amount to the optimal titer when the same amounts of DNA_chrom_ are available. **D** Heap map analysis of peaks around transcription start sites. Read intensities of the peaks around ± 5 Kb of TSSs were visualized by Partek Flow software. The library ID indicates DNA_chrom_ (µg)-antibody titer in ChIP reactions. The reactions that the optimal titer was determined in Fig. 2 were marked with orange. **E** The representative snapshot image of ChIP-seq results generated with various antibody titers and after normalizing antibody amount to the optimal titer. The read densities were visualized in a 212 kb genomic region around the *ACTB* gene using the Integrative Genomics Viewer

To understand the impact of the antibody titer on the outcome of ChIP experiments and demonstrate the utility of titration-based normalization of antibody amount per available chromatin input, we performed three independent ChIP experiments by incubating various amounts of chromatin input with a fixed amount or normalized amounts of antibody at the optimal titer (Fig. 3A). Chromatin input was prepared from 40 million fixed K562 cells and DNA_chrom_ was measured as described above. Chromatin was diluted to obtain chromatin inputs with various DNA_chrom_ values ranging from 0.3 to 20 μg. Each of these inputs was then incubated with fixed 0.25 μg of antibody (left panel) or the normalized antibody amounts by DNA_chrom_ at the optimal titer of 0.25 μg antibody/10 μg of DNA_chrom_ (T = 1, right panel). Consistent with the results in Fig. 2, ChIP reactions over 10 µg of DNA_chrom_ and the fixed amount of 0.25 µg of antibody (i.e., T = 1 or less) exhibited an enrichment exceeding 100-fold in multiple positive loci with yields below 0.5% (Fig. 3A, left panel). ChIP reactions utilizing less than 10 µg of DNA_chrom_ (i.e., T > 1) showed lower enrichments with higher yields. In contrast, normalized ChIP reactions to the optimal titer (i.e., T = 1) showed similar ChIP yields (< 0.5%) across a range of input DNA_chrom_ amounts of 0.3 to 20 µg (Fig. 3A, right panel). Furthermore, this approach clearly improved ChIP enrichment in the reactions utilizing less than 10 µg of DNA_chrom_ relative to the experiments employing a fixed amount of antibody. Similarly to the results shown in Fig. S3B (Additional file 4), the ChIP with the fixed antibody amount showed an inverse correlation between ChIP yield and enrichment (Additional file 5: Fig. S4A). However, we observed consistent ChIP yield and improved enrichment when using normalized antibody amounts. To understand the impact of antibody titer in the context of ChIP-seq, we further investigated the mapping results of libraries generated from experiments employing fixed or normalized amounts of antibody. All libraries showed similar informatics QC results including read number, library complexity, and mapping rate (Additional file 6: Table S2). The library ID indicates chromatin input amount (µg) by DNA_chrom_ -the antibody titers applied with the optimal titer marked as T1. Similarly to the results from the qPCR-based enrichment assays, peak numbers negatively correlated with the ChIP yield when fixed antibody amouns were used but remained flat when normalized antibody amounts were applied (Additional file 5: Fig. S4B). Considering all peaks, ChIP-seq datasets generated with different antibody titers (T0.5 to T32) and the normalized titer (T = 1) showed coefficients (*r*) ranging from 0.78 to 0.89 (Additional file 7: Fig. S5A and B). In ChIP reactions employing fixed antibody amount, the correlation coefficients (*r*) were variable and tended to be lower in experiments using higher antibody titers. However, *r* (i.e., the similarity among the datasets) was improved and more consistent when the normalized amount to the optimal titer was used. Consistent with the fold enrichment results obtained by qPCR in Fig. 3A, peak numbers were low in the reactions with antibody titers over 1 but improved when the antibody was applied at the optimal titer (Fig. 3B and C). To further understand the variations in peak numbers in the reactions, we analyzed signal intensities of peaks around TSSs (Fig. 3D and Additional file 7: Fig. S5C and D). Signal intensities were clearly lower when the antibody titer was over 1, but higher and more consistent when the antibody titer was less than or equal to 1. Fraction of reads in peaks (FRiP) showed a gradual decrease with increasing antibody titers but became independent of input chromatin amounts when the antibody was applied at the optimal titer (Additional file 7: Fig. S5E). Similarly, the IGV browser showed low peak intensities but relatively high background (i.e., signal at non-peak areas) in ChIP reactions with antibody titers over 1 (Fig. 3E). The libraries generated from the experiments using antibody titers less than or equal to 1 showed comparable and consistent peak intensities. These results suggest that antibody titers over the optimal ratio increase the background signal and result in a reduced number of peaks. Altogether, these observations clearly indicate that the amount of antibody related to the amount of chromatin input (i.e., the antibody titer) is critical for the outcome of ChIP experiment even when ChIP-seq validated antibodies are used, and the use of different antibody titers in ChIP reactions results in inconsistent data quality. Thus, utilizing antibodies at the optimal titer for ChIP reaction improves the experimental outcome and the consistency among samples both within and across experiments.

### Validation of the titration-based and normalized ChIP-seq approach in a large-scale project

**Fig. 4:**
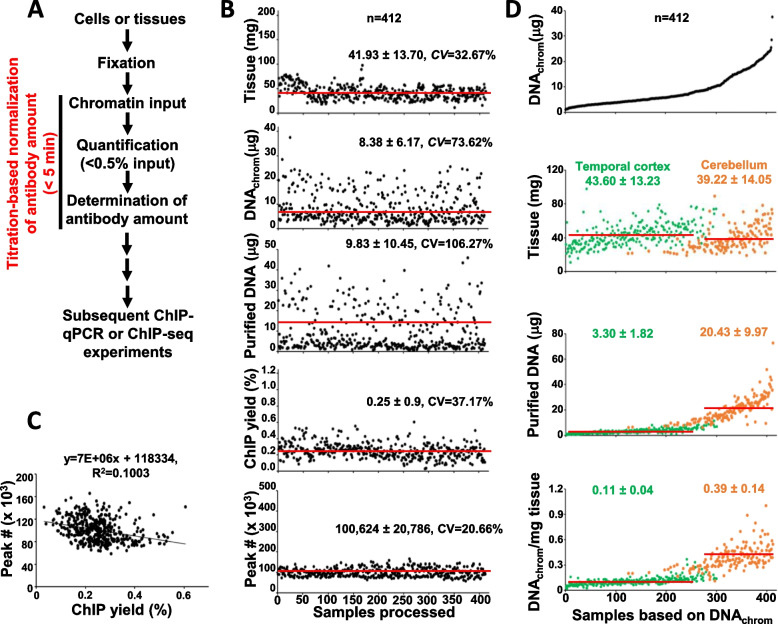
Titration-based and normalized ChIP-seq approach improves experimental consistency in a large-scale ChIP-seq project. **A** A schematic diagram of titration-based and normalized ChIP-seq. Chromatin amount is quickly quantitated by the high-sensitivity Qubit assay using less than 0.5% of input. The amount of ChIP-seq validated antibody is normalized to the optimal titer in individual ChIP reaction depending on available amount of DNA_chrom_. It takes less than 5 min for one antibody where its optimal titer is predetermined, but it may take longer for multiple antibodies **B** Titration-based and normalized approach generates consistent ChIP yield and peak number over time. Experiments were performed over 1.5-year window as described. Tissue amount (mg), chromatin amount by DNA_chrom_ and purified DNA, ChIP yield, and peak number were plotted with the order of sample processing. The similar y-axis scales with the distribution of chromatin amounts by purified DNA were used for other plots. The solid line in each plot indicates the mean value of measurement. The mean ± SD with the coefficient of variation (CV) was shown in each plot to determine the variability of collected data throughout the samples. **C** Normalization of antibody titer minimizes the negative correlation of ChIP yield with peak number. Peak number was plotted with % ChIP yield. The solid line indicates the trend line of linear regression. The equation is shown the top. **D** DNA_chrom_ correlates with DNA amount/mg of tissue. Collected data was sorted based on DNA_chrom_ and plotted with tissue amount (mg), chromatin amount by purified DNA, and DNA amount/mg of tissue. Each dot represents the individual sample from temporal cortex (green) and cerebellum (orange)

Next, we demonstrated the utility of our titration-based and normalized ChIP-seq approach in a study involving 412 post-mortem human cerebellum (CER) and temporal cortex (TCX) brain tissue samples (Fig. 4A). The experiments were performed over 1.5 years using one ChIP-seq-validated H3K27ac antibody to minimize technical variations by the antibody itself. We closely followed up tissue weights, sizes of chromatin input, amounts of chromatin input determined by DNA_chrom_ and purified DNA, ChIP yields, fold enrichments of H3K27ac-positive genomic loci over a negative locus, quality control matrices of sequenced reads, and peak numbers. The cellularities of individual samples were not available. The sizes of DNA isolated from chromatin inputs was similar, ranging from 100 to 300 bp throughout the samples (Additional file 8: Fig. S6). The additional information obtained from individual samples is available (Additional file 9: Table S3). We analyzed the variability of the collected data in the order of sample processing over time by determining the coefficient of variation (CV) [23, 24]. Chromatin input was prepared from tissue amounts ranging from 11.0 to 97.7 mg (CV = 32.67%) (Fig. 4B, top panel). DNA_chrom_ was measured using 0.4% of chromatin input. DNA_chrom_ (2^nd^ panel) was closely correlated with the chromatin amount determined by purified DNA (3^rd^ panel). Overall, the amounts of chromatin input were highly variable compared to tissue amounts with CV values of 73.62% from DNA_chrom_ (8.38 µg (mean) ± 6.17 (SD)) and 106.27% from the chromatin amount (9.83 µg ± 10.45) determined by purified DNA. The ChIP yield was less variable (CV = 37.17%) (3^rd^ and 4^th^ panels). All samples generated more than 0.65 ng of ChIP DNA, which was sufficient for library preparation. The detailed mapping results for individual libraries were included (Additional file 10: Table S4), showing that the number of sequenced reads, library complexity, and percent mapping rate is similar and acceptable as indicated by ENCODE guidelines. Peak numbers were highly consistent (CV = 20.66%; average number of peaks: 100,624) throughout samples in recurring experiments over the duration of the study (bottom panel). To obtain further insight into experimental outcome and data quality, we sorted data by tissue weight, chromatin amount determined by purified DNA, ChIP yield, and peak number (Additional file 11: Fig. S7). Consistent with the results arranged by the order of sample processing over time (Fig. 4B), all sorting-based analysis indicated that the titration-based and normalized approach generates consistent ChIP yield and peak number regardless of tissue and chromatin input amount. Next, we compared the distribution of peak numbers among samples with publicly available large-scale H3K27ac ChIP-seq datasets [25]. The titration-based and normalized ChIP-seq approach yielded higher mean peak number with less standard deviation (100,623 peaks (mean) ± 20,786 (SD) in this study vs 77,390 ± 52,468 in monocytes, 57,126 ± 29,041 in neutrophils, or 43,911 ± 31,663 in CD4^+^ T-cells) and better consistency (CV = 20.65% (this study) vs 67.80% (monocytes), 50.84% (neutrophils), 72.11% (CD4^+^ T-cells)) (Additional file 12: Fig. S8). It is noteworthy that the lowest peak number was 58,426 in our study and none of the experiments performed in the 412 samples failed. Similarly to the results obtained in K562 cells (Fig. 3), the results from 412 brain tissues indicated consistent ChIP yield and peak numbers with no patterns of inverse correlation (Fig. 4C). These results demonstrate that the titration-based and normalized ChIP approach generates reproducible and reliable data with less variation in peak numbers among samples. In addition, we found that DNA_chrom_ was more closely correlated with DNA amount/mg tissue than with tissue amounts (Fig. 4D). The samples were sorted by DNA_chrom_, and the distribution of samples was investigated following tissue origins, TCX and CER. It was reported that the CER region of brain has higher cell density (i.e., cellularity) compared with the TCX region [26, 27]. DNA_chrom_ was not well correlated with tissue amounts across all samples but we found weak correlation when the TCX (green dots) or CER (orange dots) samples were separately analyzed (2^nd^ panel). The majority of the TCX samples had lower DNA_chrom_ levels despite the higher amounts of tissue available for experiment. A similar pattern was observed when chromatin amounts were determined by purified DNA (3^rd^ panel). In contrast, CER samples yielded more chromatin as determined either by DNA_chrom_ (1^st^ panel) or purified DNA (3^rd^ panel). As the DNA amount/mg tissue is dependent on the number of nucleated cells (i.e., cellularity), we speculated that DNA_chrom_ potentially provides a measure of relative cellularity among samples. Consistent with this notion, the distribution of DNA_chrom_/mg tissue was well segregated into TCX and CER samples (bottom panel). Consistent with cell density patterns, we found DNA_chrom_/mg tissue values to be 3.54 fold higher in CER (0.39 mg ± 0.04) compared to TCX (0.11 mg ± 0.04). Alltogether, the titration-based normalization approach shows consistent ChIP yields and peak numbers regardless of the size of the starting samples and the amounts of the extracted chromatin input, enabling improved experimental consistency in ChIP applications performed over extended periods of time.

## Discussion

ChIP is an antibody-based approach to study regulatory factors and epigenetic modifications within a chromatin template. Fragmented chromatin encompassing around 0.1 – 1 kb of DNA, typically generated by sonication, MNase, or a combined approach, is incubated with a ChIP-validated antibody against a target of interest in immunoprecipitation reactions [7, 8]. Observing the limitations on specificity and availability of antibody, the ENCODE guidelines clearly defined the experimental requirements regarding antibody validation, antibody requirements in ChIP experiments, and documentation of the antibody for publication purposes [9, 10]. Our results clearly indicated that ChIP efficiency and outcome are heavily dependent on the conditions used in the ChIP reaction even for ChIP-validated antibodies. In this study, we systemically and specifically focused on the impact of chromatin input amount, one of the most variable and unpredictable factors in these experiments, on ChIP efficiency and outcome. Consistent with the observations in protein extract-based immunoprecipitation [17], chromatin-based immunoprecipitation similarly shows a clear inverse correlation between yield and specificity (i.e., enrichment) (Fig. 3A and Additional file 5: Fig. S4A and C). The ChIP-seq validated and frequently utilized anti-H3K27ac antibody (Abcam, Cat. ab4729) showed high variation in ChIP yield, fold enrichment, and peak number depending on the antibody titer used in the ChIP reaction. However, ChIP efficiency is consistent and becomes less variable when the antibody amount is normalized to the optimal titer (Fig. 3 and Additional file 5: Fig. S4B and D). These results indicate that the antibody titer in a ChIP reaction is critical for ChIP efficiency and assay outcome. It is reasonable to include the antibody titer (i.e., the ratio of antibody amount/chromatin input amount) in antibody validation experiments or publication of ChIP-based results as a quality control for the ChIP experiment.

The quick and sensitive quantification method of chromatin input immediately after its preparation is a prerequisite for determining the optimal amount of antibody in each individual ChIP reaction. We utilized the Qubit assay to directly measure DNA content in chromatin input (DNA_chrom_) and demonstrated DNA_chrom_ is highly correlated with chromatin amount determined by purified DNA in a large number of samples originating from distinct sample types including cells and tissues (Fig. 1). It is noteworthy the measurement takes less than 5 min and requires only 0.2—2% of total input (Figs. 1 and 4A). In general, we observed that the difference between chromatin amounts determined by DNA_chrom_ and purified DNA from individual sample type was linear within a broad range. However, DNA_chrom_ was measured as low as about 20% in most of sample types but about 50% in samples derived from peripheral blood, compared with the amount of purified DNA (Fig. 1 and Additional file 2: Fig. S1). We speculate that the Qubit reagent reacts with dsDNA within native chromatin less efficiently than in purified form, and the reactivity is dependent on chromatin compactness. Consistent with this hypothesis, cells in peripheral blood are known to have highly compacted chromatin [28, 29]. Despite the lower reactivity of the Qubit reagent in chromatin input derived from blood-derived samples, we still believe DNA_chrom_ can be used for titration-based normalization of antibody close to the optimal titer in individual ChIP reaction. Interestingly, DNA_chrom_ showed a better correlation with DNA amount/mg of tissue compared to tissue amount, indicating that tissue cellularity, along with the quantity of tissue, contributes to the yield of chromatin input. These observations suggest that DNA_chrom_ allows for measurement of the relative cellularity of individual experimental tissues that would otherwise be ignored. Further studies are needed to validate DNA_chrom_/mg of tissue as a valid indicator of tissue cellularity (i.e., the number of nucleated cells in a given tissue).

The typical downstream analysis of ChIP-seq data is related to identifying genomic locations associated with the target of interest in each sample, and differential binding analysis between samples [9, 10, 30, 31]. ChIP-seq libraries are generated in single or recurring experiments assuming ChIP efficiency is similar among samples or throughout experiments. However, it is challenging to normalize ChIP efficiency across samples if the chromatin input from individual samples is highly variable (Fig. 4C), or if experiments are performed over an extended period of time [32, 33]. Consequently, the variation in ChIP efficiency may lead to unpredictable and variable outcomes, especially in peak number after peak calling, as indicated in Fig. 3. A wide range of peak numbers makes differential binding analysis difficult or limited, resulting in the potential loss of meaningful data. Furthermore, this may impact the ability to perform comparative analyses of ChIP-seq data from different research groups [18, 34]. Therefore, we strongly believe that normalizing antibody titer per available chromatin amount from the individual sample is a more robust method to experimentally normalize ChIP efficiency throughout samples, leading to improved experimental quality and consistency in ChIP applications.

## Conclusions

The amount of chromatin input available in ChIP reaction is often unpredictable. We describe a simple, quick, and sensitive method to estimate the amount of solubilized chromatin input immediately after its preparation, and we demonstrated that titration-based normalization of antibody amount in ChIP reaction improves experimental consistency and overall data quality in a large-scale ChIP-seq project. Aligned with continuing updates on the ENCODE guideline to standardize ChIP experimental conditions, we anticipate that this approach will improve experimental and data consistency in ChIP experiments.

## Methods

### Cell culture and reagents

K562 cells were purchased from ATCC and grown in IMDM (Iscove's Modified Dulbecco's Media) at 37 °C and 5% CO_2_. The media contained 10% calf bovine serum and 1% Penicillin/Streptomycin.

### Preparation and direct quantification of chromatin input in K562 cells

Chromatin input was prepared from fixed K562 cells and the amount of solubilized chromatin was indirectly measured from purified DNA after the cross-linking reversal as previously described [19]. DNA content in chromatin input (DNA_chrom_) was directly measured by the Qubit dsDNA High Sensitivity assay (Invitrogen, Q32851) [20, 21] by incubating 2 µl of chromatin input with 198 µl of Qubit reagent and calculated following the manufacturer’s instructions as demonstrated for purified DNA. We tested the following amounts of chromatin input in the experiments (*n* = 3): 0.3, 0.625, 1.25, 2.5, 5.0, 10, and 20 µg. The buffer composition of chromatin input includes 60 mM Tris–HCl, 108 mM NaCl, 30 mM KCl, 0.5 mM CaCl_2_, 10 mM EDTA, 1% Triton X-100, 0.1% sodium deoxycholate. DNA_chrom_ was compared with the amount of chromatin input measured by purified DNA.

### *Quantification of chromatin input by DNA*_*chrom*_* in various sample types*

Chromatin was prepared from cells and tissue sampes in the context of ChIP experiments as previously described [19, 35]. DNA_chrom_ was measured from 0.5—1% of chromatin input in individual ChIP reaction as described above. Total 666 indiffendent samples were analyzed including 8 different cell lines (*n* = 78), 3 different solid tissues (*n* = 533), and 2 different samples derived from peripheral blood (*n* = 54). The detailed information on individual samples is summarized (Additional file 1: Table S1). The amounts of chromatin input determined by DNA_chrom_ and purified DNA were compared using the linear regression model. It is noteworthy users should test that their ChIP buffer does not interfere with Qubit measurements.

### Analysis of ChIP enrichment by real-time PCR

Real-time PCR analysis was performed using SYBR Green universal PCR mixes (Bio-Rad) following the manufacturer’s instructions. The following primers were used in the experiments: H3K27ac-positive control locus: *SMARCA4*-TSS-F: 5’-TTGGCGAAGCTGCGATCGGG-3’, *SMARCA4*-TSS-R: 5’-AGGGGACCGCTAATGCCCGT-3’; *PABPC1*-TSS-F: 5’-CACTCTCAGCACTAACCGCC-3’, *PABPC1*-TSS-R: 5’-CGGCGCGGGGTATAAGTAGA-3’; *PABPC1*-0.5 kb-F: CAGCGGCAGTGGATCGA, *PABPC1*-0.5 kb-R: 5’-GGACAAAAATCAACCGGAATTG-3’; *ACTB*-TSS-F: 5’-CCTCATGGCCTTGTCACAC-3’, *ACTB*-TSS-R: 5’-GCCCTTTCTCACTGGTTCTCT-3’; *GAPDH*-TSS-F: 5’-CCCACTCCTCCACCTTTGAC-3’; *GAPDH*-TSS-R: 5’-CCCAGCCACATACCAGGAAA-3’. H3K27ac-negative control locus: Ch19-intergenic-F: 5’-AGCTTGTCTTTCCCAAGTTTACTC-3’, Ch19-intergenic-R: 5’-TAGCTGTCGCACTTCAGAGGA-3’; *MYT1*-TSS-F: 5’-CCTGCCGTGTGCTGTTTTT-3’, *MYT1*-TSS-R: 5’-CACAACATGTCCCCTGGAATC-3’. We calculated the signal-to-noise ratios in multiple H3K27ac-positive loci by dividing the % enrichment of each positive locus normalized to 1% input DNA against with the % enrichment of representative negative locus in individual ChIP or library DNA.

### *Determination of optimal titer using DNA*_*chrom*_

Chromatin input was prepared from 40 million of fixed K562 as described in the above. DNA_chrom_ was directly measured by the Qubit High Sensitivity assay. The chromatin amount equivalent to 10 µg of DNA_chrom_ was incubated with different amounts of anti-H3K27ac antibody (Abcam, ab4729, lot# GR3357415-1) ranging 0.05—10.0 µg overnight. All other steps were followed as published [19]. Purified input and ChIP DNAs were measured by the Qubit High Sensitivity assay and the percentage of ChIP yields were calculated by dividing the amount of ChIP DNA by the amount of DNA from total input chromatin. The profiles of input DNAs were analyzed by the Fragment Analyzer (Advanced Analytical Technologies; AATI; Ankeny, IA) using the High Sensitivity NGS Fragment Analysis Kit (Cat. #DNF-486). The enrichment was determined by qPCR using target-specific primer as described above. The results were analyzed by nonlinear regression model using Prism GraphPad, and presented as mean ± SD. We determined the optimal titer as the the ratio between antibody and chromatin amounts to yield 1 – 5 ng of ChIP DNA and 5 – 200 fold enrichment in multiple positive over negative loci. We included a schematic diagram showing step-by-step methods to define ideal titer of a ChIP-seq validated antibody (Additional file 3: Fig. S2).

### Validation of antibody titers in varying amounts of chromatin input

Chromatin inputs were prepared from 40 million of fixed K562 cells, and DNA_chrom_ was directly measured by the Qubit High Sensitivity assay. Chromatin amount was aliquoted into 312.5, 625, 1,250, 2,500, 5,000, 10,000 and 20,000 ng in final 1 ml of ChIP buffer. 0.25 µg of ChIP-seq validated anti-H3K27ac antibody (Abcam, ab4729) was added to create the ChIP reactions with various antibody titers (the ratios between antibody amount per chromatin amount). Or, normalized amounts of the antibody to the optimal titer were added into ChIP reactions and incubated overnight. ChIP yield and fold enrichment were analyzed as described above. The ChIP-seq libraries were prepared using the ThruPLEX DNA-seq kit (Takara, Cat# R400675) according to the manufacturer’s instructions. The profiles of input and library DNAs were analyzed by the Fragment Analyzer (Advanced Analytical Technologies) using the High Sensitivity NGS Fragment Analysis Kit (Cat. #DNF-486). Enrichment was analyzed in ChIP and library DNAs by performing qPCR in the genomic loci targeting the TSSs of an active (i.e., *SMARCA4*-TSS or *ACTB*-TSS) or inactive gene (i.e., *MYT1*-TSS) and an intergenic region (Ch19-intergenic). The ChIP-seq libraries were sequenced to 51 base pairs from both ends on an Illumina HiSeq 4000 instrument in the Mayo Clinic Center for Individualized Medicine Medical Genomics Facility. Raw sequencing reads were processed and analyzed using the ChIP-seq analysis pipeline in Partek® Flow® software, v10.0. (https://www.partek.com/partek-flow/) (Partek, Inc., St. Louis, MO) to obtain visualization files and a list of peaks. Briefly, paired-end reads were mapped to the human reference genome (hg19) with default settings, and only pairs with at least one of the ends being uniquely mapped were retained for further analysis. Peaks were called using the MACS2 algorithm [36] at FDR <  = 1% and were visualized. The signal intensities over peak center at transcription start sites were calculated for all peaks with the smallest FDR. The between-sample correlation coefficient was calculated for all the peaks merged from all peaks in each sample and was plotted by deepTools2 [37]. FRiP score [9], i.e., the fraction of reads in blacklist-filtered peaks over total usable reads, was calculated for each library following the ENCODE guideline (https://www.encodeproject.org/atac-seq/).

### Titration-based and normalized ChIP-seq in frozen post-mortem human brain tissues

Post-mortem human cerebellum (CER, *n* = 153) and temporal cortex (TCX, *n* = 247) brain tissue samples were obtained from the Mayo Clinic Brain Bank (Mayo Clinic Florida, Jacksonville) and the Banner Sun Health Institute (Sun City, AZ). Samples were randomized into experimental batches according to tissue region, brain bank, and known biological variables. For each experiment, the average of 10 frozen tissue samples was processed immediately after measuring the weight of individual sample. Tissues were homogenized for 30 s in PBS using tissue grinder (ACTGene, ACT-AG 3080). Homogenized tissues were cross-linked to final 1% formaldehyde, quenched with 125 mM glycine, and washed with TBS. The fixed homogenates were resuspended in cell lysis buffer (10 mM Tris HCl, pH7.5, 10 mM NaCl, 0.5% IGEPAL) and incubated on ice for 10 min. The lysates were washed with MNase digestion buffer (20 mM Tris–HCl, pH7.5, 15 mM NaCl, 60 mM KCl, 1 mM CaCl_2_) and were incubated in the fresh 250 μL MNase digestion buffer containing proteinase inhibitor cocktails in the presence of 500 gel units of MNase (NEB, Cat.# M0247S) at 37 °C for 20 min with continuous mixing in thermal mixer. After adding 250 µL of 2X Stop/ChIP/Sonication buffer (100 mM Tris–HCl, pH8.1, 20 mM EDTA, 200 mM NaCl, 2% Triton X-100, 0.2% Sodium deoxycholate), the lysates were sonicated for 20 min (30 s on / 30 s off) using Diagenode Bioruptor pico and centrifuged at 21,130 xg for 10 min. The supernatant was transferred to a new tube and chromatin amount was directly measured by the Qubit dsDNA High Sensitivity assay using 0.1% of total input. Antibody amount was determined by the chromatin amount of individual sample following the titer (4.3 µl of H3K27Ac antibody (CST cat # 8173, lot 1)) per 20 µg of chromatin as previously published [35]. SDS (0.05% final conc.) was added to the reaction and 1% of the input was saved. The reaction was Incubated overnight at 4 °C in a rotator. After adding 30 µL of protein G-magnetic beads, the reactions were further incubated for 3 h. The beads were extensively washed with ChIP buffer (50 mM Tris–HCl, pH8.1, 10 mM EDTA, 100 mM NaCl, 1% Triton X-100, 0.1% sodium deoxycholate), high salt buffer (50 mM Tris–HCl, pH8.1, 10 mM EDTA, 500 mM NaCl, 1% Triton X-100, 0.1% sodium deoxycholate), LiCl_2_ buffer (10 mM Tris–HCl, pH8.0, 0.25 M LiCl_2_, 0.5% NP-40, 0.5% Sodium deoxycholate, 1 mM EDTA), and TE buffer. Bound chromatins were eluted and reverse-crosslinked at 65 °C overnight. DNAs were purified using Min-Elute PCR purification kit after the treatment of RNase A and proteinase K. Purified input and ChIP DNAs were measured by the Qubit dsDNA High Sensitivity assay. ChIP-seq libraries were prepared using the ThruPLEX DNA-seq kit (Takara, Cat# R400675) according to the manufacturer’s instructions. The profiles of input and library DNAs were analyzed by the Fragment Analyzer (Advanced Analytical Technologies; AATI; Ankeny, IA) using the High Sensitivity NGS Fragment Analysis Kit (Cat. #DNF-486). Enrichment was analyzed in library DNAs by performing qPCR in the genomic loci targeting the TSSs of an active (*SMARCA4*-TSS and *ACTB*-TSS) or H3K27ac-negative intergenic region (Ch19-intergenic). The ChIP-seq libraries were sequenced to 51 base pairs from both ends on an Illumina HiSeq 4000 instrument in the Mayo Clinic Center for Individualized Medicine Medical Genomics Facility. Raw sequencing reads were processed and analyzed using the HiChIP pipeline [38] to obtain the quality control metrics of sequenced reads following the ENCODE guidelines, visualization files, and a list of peaks. Paired-end reads were mapped to the human reference genome (hg38) by BWA [39] with default settings, and only pairs with at least one of the ends being uniquely mapped were retained for further analysis. Peaks were called using the MACS2 algorithm at FDR <  = 1% using randomly pooled reads (total 22.8 M PE reads, 1.09 M PE reads per CER library and 653,000 reads per TCX library) from 21 CER- or TCX-derived input libraries sequenced in the early phase of project [40, 41].

## Supplementary Information

**Additional file 1: Table S1.** Detailed information of DNA_chrom_ and the amount of purified DNA in various sample types.

**Additional file 2: Figure S1.** DNA_chrom_ shows a linear correlation with the amounts of chromatin input measured by purified DNA in individual sample type. DNA amount was directly measured in chromatin input by the Qubit dsDNA high sensitivity assay and compared with the amount of purified DNA. R^2^ was calculated by the linear regression model. A: Cell lines (*n*=78) include 9 different cultured cell lines as indicated in the bottom table. B-D: Solid tissues (*n*=534) include samples from human glioma tumor (B), samples from human anaplastic thyroid cancer (C), and samples from post-mortem human brain tissues (D). E-F: The samples (*n*=54) derived from peripheral blood include buffy coat (E) and PBMC (F).

**Additional file 3: Figure S2. **A schematic diagram showing step-by-step methods to define ideal titer of a ChIP-seq validated antibody.

**Additional file 4: Figure S3.** ChIP yield negatively correlates with the enrichment of positive targets. A: The profiles of DNA size purified from chromatin input and ChIPed chromatin. DNA was purified from input chromatin and ChIPed chromatin-antibody complexes described in Fig. 2 and analyzed by the Fragment Analyzer. The representative image was presented. The ratio between the antibody amount and DNA_chrom_ at the optimal titer was indicated as titer 1 (T1) and the titers were shown on the top. B: The chromatin amount equivalent to 10 µg of DNA_chrom_ was subjected to immunoprecipitation using various amounts of anti-H3K27ac antibody ranging 0.05 - 10.0 µg. ChIP yield was plotted with the fold enrichment of H3K27ac-positive *PABPC1*-TSS over H3K27ac-negative *MYT1*-TSS loci in individual reactions. Three independent experiments were performed, and the results were presented by the linear regression model. C: Fold enrichment varies depending on positive and negative genomic loci used. The fold enrichments of positive over negative genomic loci were accessed by ChIP-qPCR. Transcription start sites (TSSs) of *ACTB* or *GAPDH* are considered as H3K27ac-positive. *MYT1*-TSS is considered as H3K27ac-negative (left panel). Similar analysis was done using an intergenic region (C19 intergenic) nearby the *ACTB* gene as H3K27ac-negative locus (right panel). The optimal titer (T1) is shown as orange arrow.

**Additional file 5: Figure S4. **Titration-based normalization of antibody amount to the optimal titer leads to consistent ChIP yield and improved data quality. A-D: Fixed 0.25 µg of anti-H3K27ac antibody or normalized amounts to the optimal titer were immunoprecipitated with DNA_chrom_ ranging 0.31 – 20 µg in ChIP reactions. ChIP yield was compared with the fold enrichments of H3K27ac-positive PABPC1-TSS over H3K27ac-negative *MYT1*-TSS loci in individual reactions of fixed antibody amount (A) and normalized amounts to the optimal titer (B). ChIP yield was compared with peak numbers from individual reactions of fixed antibody amount (C) and normalized amount to the optimal titer (D). The result was presented by the linear regression model.

**Additional file 6: Table S2. **Mapping results of ChIP-seq libraries used in Fig. 3. Raw sequencing reads were processed and analyzed using the ChIP-seq analysis package in the Partek Flow software. BWA aligner was used for mapping to the human reference genome (hg19). Peaks were called using the MACS2 algorithm at FDR <= 1%.

**Additional file 7: Figure S5. **The quality and consistency of ChIP-seq libraries generated by the titration-based normalization approach.  A-B: Correlation analysis between ChIP-seq datasets generated from different antibody titers. The Pearson’s correlation coefficient was visualized for the libraries generated from the conditions with the fixed amount of antibody (A) and normalized antibody amount to the optimal titer (B) in ChIP reactions. The library ID indicates DNA_chrom_ (µg)-antibody titer in ChIP reactions. The libraries generated from 10 µg of DNAchrom was highlighted as green. C-D: Read intensities in promoter-associated peaks. Read count was visualized at peaks around TSSs for the libraries generated from fixed (C) or normalized (D) antibody amount. E: Fraction of reads in peaks (FRiP) is negatively correlated with antibody titer but improved when the antibody amount is normalized to the optimal titer. The score of FRiP was calculated by following the ENCODE guideline and visualized with DNAchrom and antibody titer. The libraries generated from 10 µg of DNAchrom was indicated as orange arrow.

**Additional file 8: Figure S6. **The profiles of purified DNA from chromatin input. DNA was purified from input chromatin after cross-linking reversal, RNase treatment, and proteinase K treatment. Purified DNA was analyzed by the Fragment analyzer (FA). The number indicates sample ID with the order of sample processing. Note: the FA analysis of purified input DNAs from the samples 69 - 75 was failed but we assumed the input profile is similar based on the sizes of their library DNAs.

**Additional file 9: Table S3. **Experimental results from 412 post-mortem human brain tissues. Tissue amount (mg), chromatin amount by DNA_chrom_ and purified DNA, ChIP yield, fold enrichments of H3K27ac-positive genomic loci over a negative locus, and peak number were collected with the order of sample processing.

**Additional file 10: Table S4. **Mapping results of ChIP-seq libraries generated from 412 post-mortem human brain tissues.

**Additional file 11: Figure S7. **The analysis after sorting shows the consistency of experimental outcome. A-D: Collected data was sorted following tissue weight (mg) (A), chromatin input by purified DNA (µg) (B), ChIP yield (%) (C), and peak number (D), and the individual sorted data were compared with other datasets.

**Additional file 12: Figure S8. **Comparison of peak number throughout the samples in large-scale H3K27ac ChIP-seq projects. A: The average peak numbers from this study and publicly available projects. The peaks were called by MACS2 using FDR<0.01. The peak number from the libraries with failed QC matrix was considered as 0. The peak number was presented as mean ± SD in box plot. B: The coefficient of variation (CV) was shown in each plot to determine the variability of results throughout the samples. The similar y-axis scales as the distribution of peak number from monocytes were used for other plots. The peak number from the libraries with failed QC matrix was considered as 0. The solid line in each plot indicates the mean value of peak number and the dashed lines indicate standard deviation (SD).

## Data Availability

The ChIP-seq datasets generated in K562 cells have been deposited in the Gene Expression Omnibus (GEO) under the accession number GSE200404.

## Abbreviations

ChIP: Chromatin immunoprecipitation
ChIP-seq: Chromatin immunoprecipitation followed by sequencing
DNA_chrom_: DNA content in chromatin input
ENCODE: Encyclopedia of DNA Elements
FRiP: Fraction of reads in peaks
CV: Coefficient of variation
