# Supplementary figures and images for: Titration-based normalization of antibody amount improves consistency of ChIP-seq experiments
Source: BMC Genomics. 2023 Apr 4;24:171. doi: 10.1186/s12864-023-09253-0 (PMC10074837; doi:10.1186/s12864-023-09253-0)

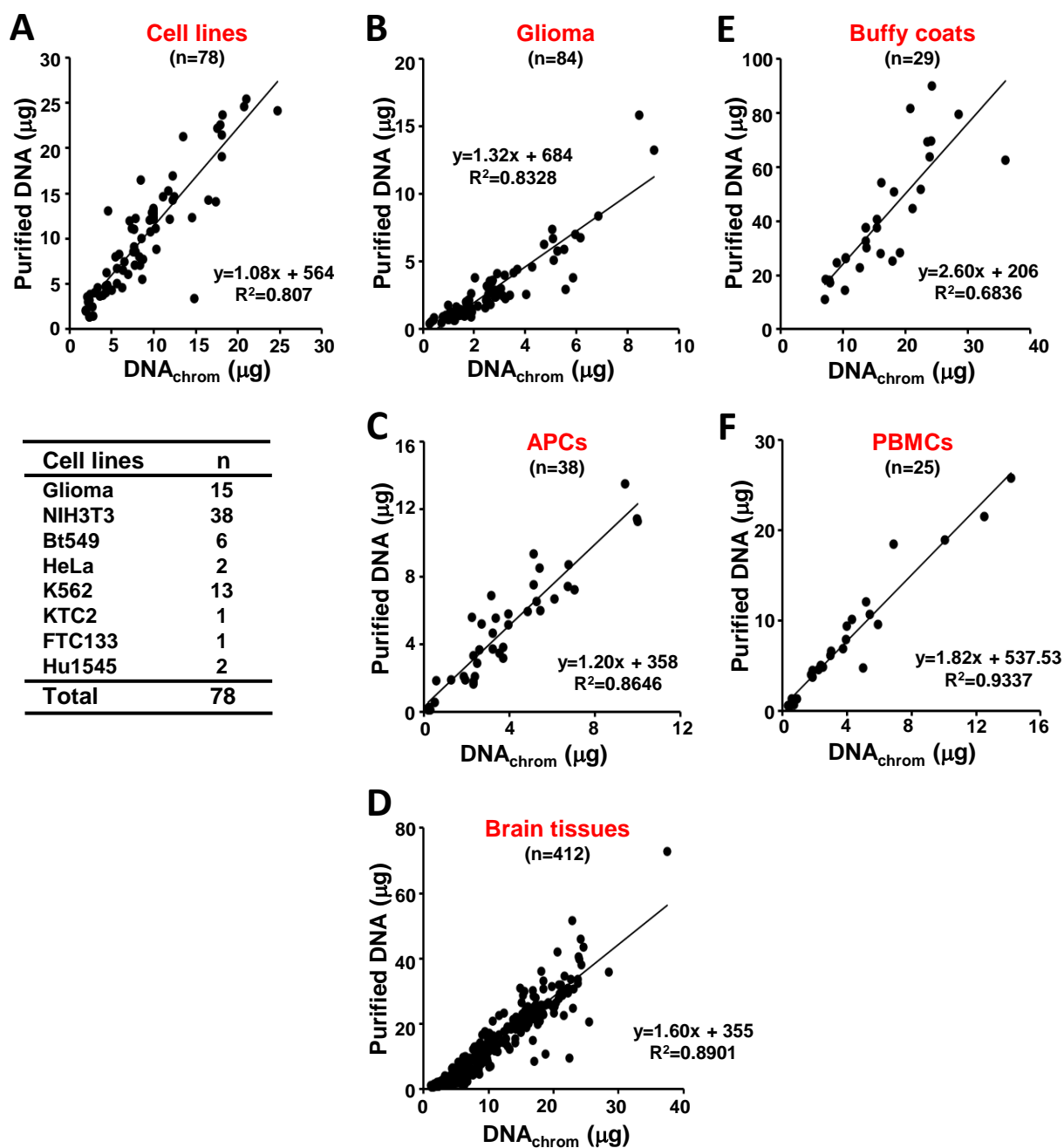

Supplement: Supplementary file 2 — Additional file 2: Figure S1. DNAchrom shows a linear correlation with the amounts of chromatin input measured by purified DNA in individual sample type. DNA amount was directly measured in chromatin input by the Qubit dsDNA high sensitivity assay and compared with the amount of purified DNA. R2 was calculated by the linear regression model. A: Cell lines (n=78) include 9 different cultured cell lines as indicated in the bottom table. B-D: Solid tissues (n=534) include samples from human glioma tumor (B), samples from human anaplastic thyroid cancer (C), and samples from post-mortem human brain tissues (D). E-F: The samples (n=54) derived from peripheral blood include buffy coat (E) and PBMC (F). [file 12864_2023_9253_MOESM2_ESM.pdf]

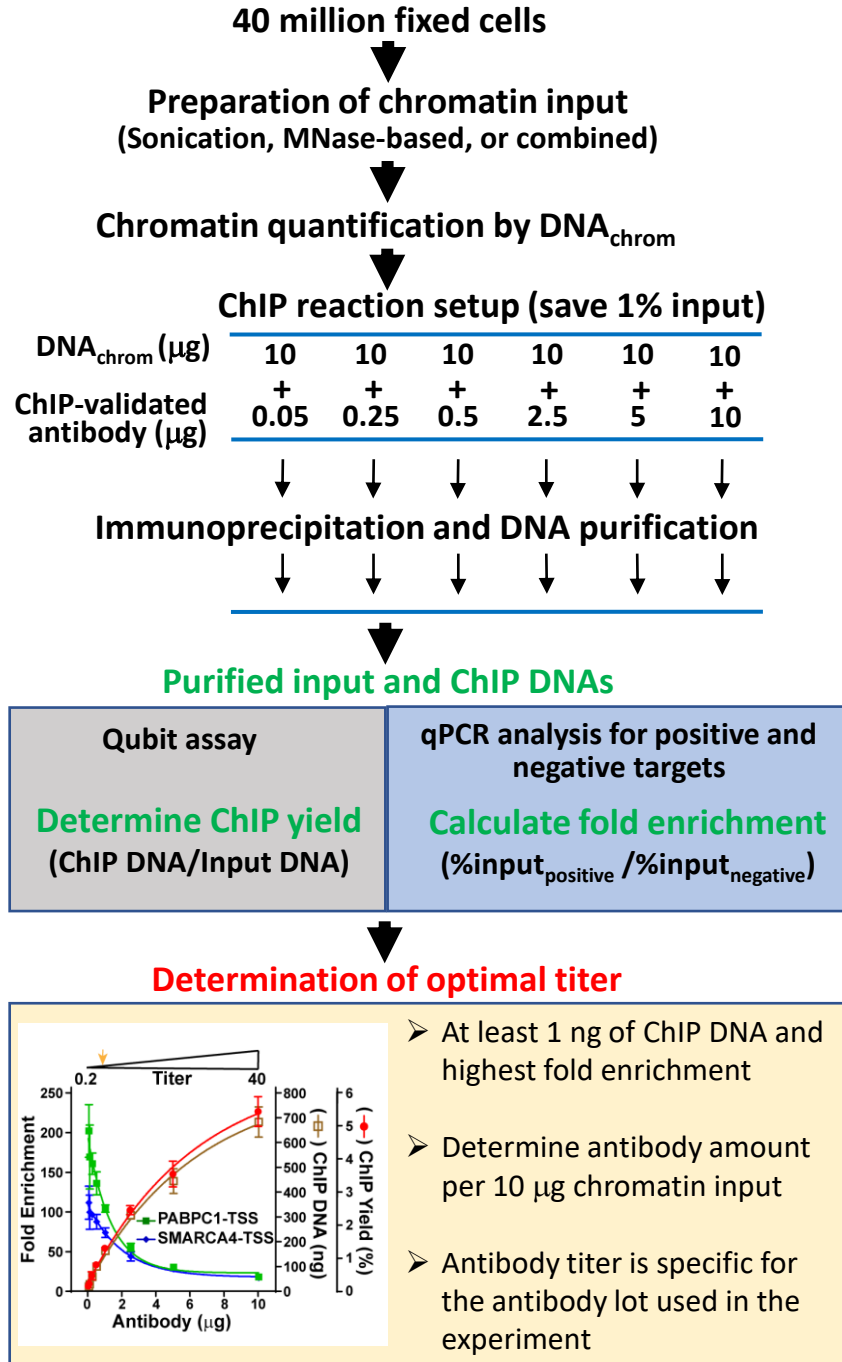

Supplement: Supplementary file 3 — Additional file 3: Figure S2. A schematic diagram showing step-by-step methods to define ideal titer of a ChIP-seq validated antibody. [file 12864_2023_9253_MOESM3_ESM.pdf]

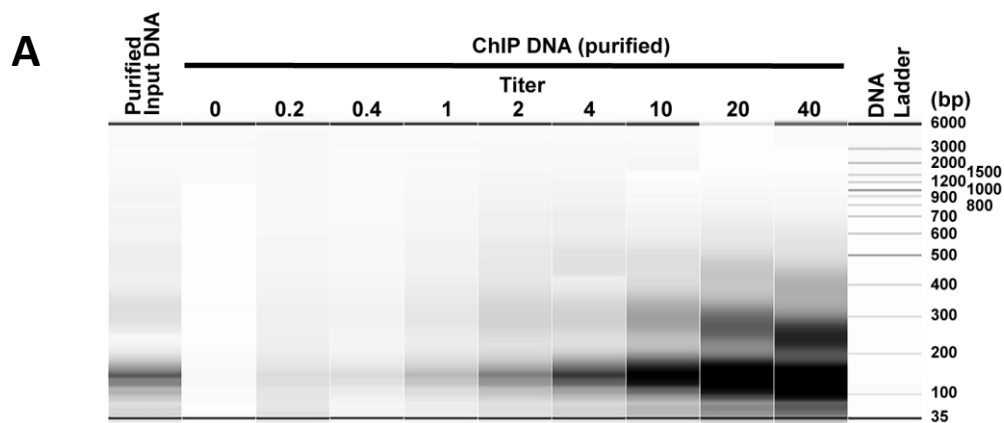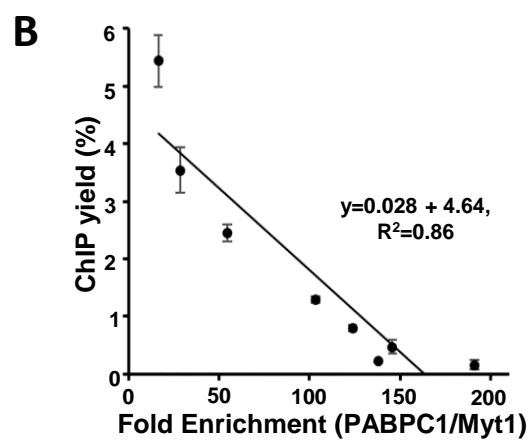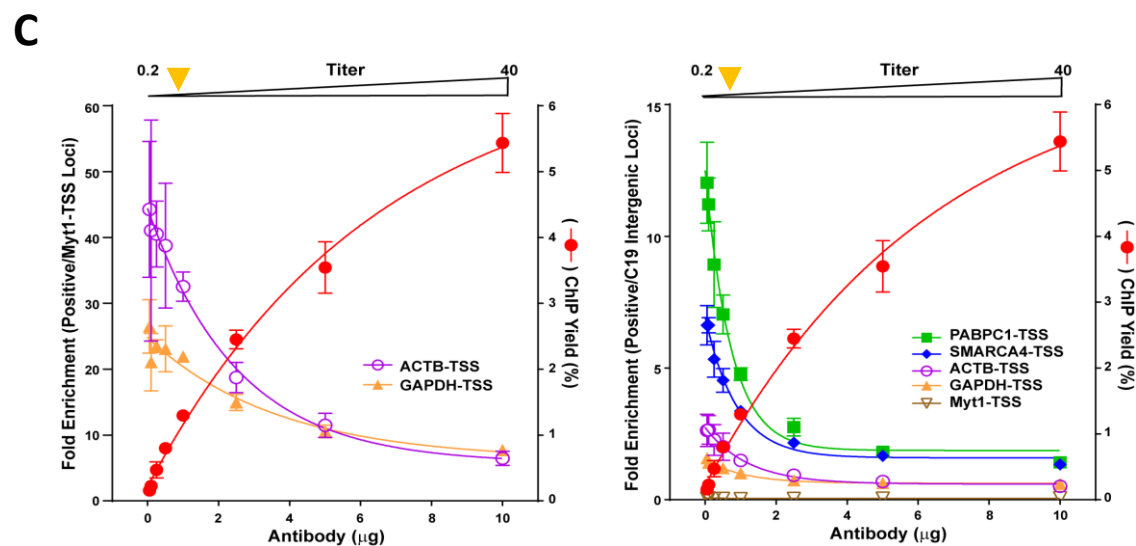

Supplement: Supplementary file 4 — Additional file 4: Figure S3. ChIP yield negatively correlates with the enrichment of positive targets. A: The profiles of DNA size purified from chromatin input and ChIPed chromatin. DNA was purified from input chromatin and ChIPed chromatin-antibody complexes described in Fig. 2 and analyzed by the Fragment Analyzer. The representative image was presented. The ratio between the antibody amount and DNAchrom at the optimal titer was indicated as titer 1 (T1) and the titers were shown on the top. B: The chromatin amount equivalent to 10 µg of DNAchrom was subjected to immunoprecipitation using various amounts of anti-H3K27ac antibody ranging 0.05 - 10.0 µg. ChIP yield was plotted with the fold enrichment of H3K27ac-positive PABPC1-TSS over H3K27ac-negative MYT1-TSS loci in individual reactions. Three independent experiments were performed, and the results were presented by the linear regression model. C: Fold enrichment varies depending on positive and negative genomic loci used. The fold enrichments of positive over negative genomic loci were accessed by ChIP-qPCR. Transcription start sites (TSSs) of ACTB or GAPDH are considered as H3K27ac-positive. MYT1-TSS is considered as H3K27ac-negative (left panel). Similar analysis was done using an intergenic region (C19 intergenic) nearby the ACTB gene as H3K27ac-negative locus (right panel). The optimal titer (T1) is shown as orange arrow. [file 12864_2023_9253_MOESM4_ESM.pdf]

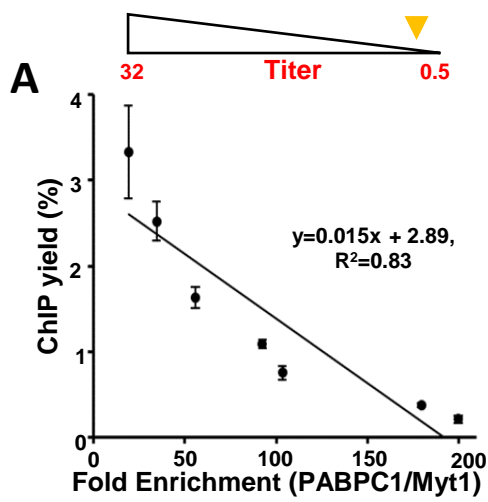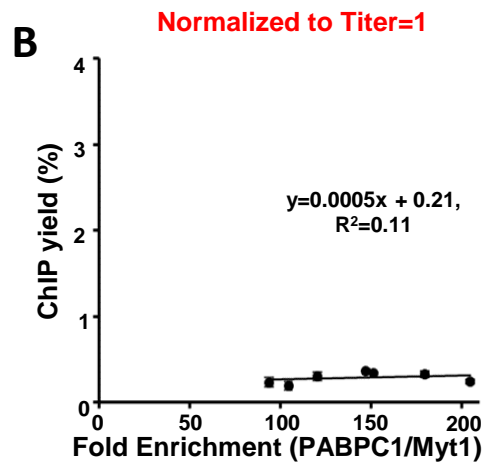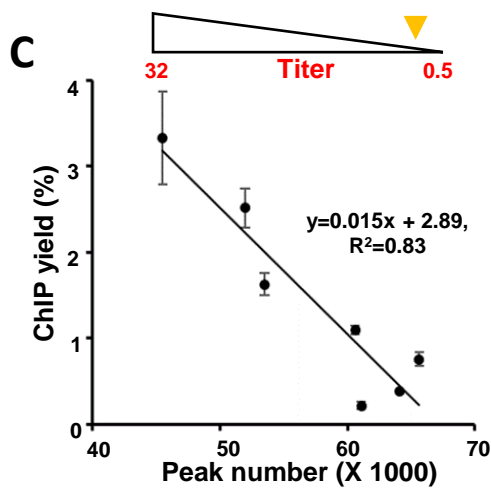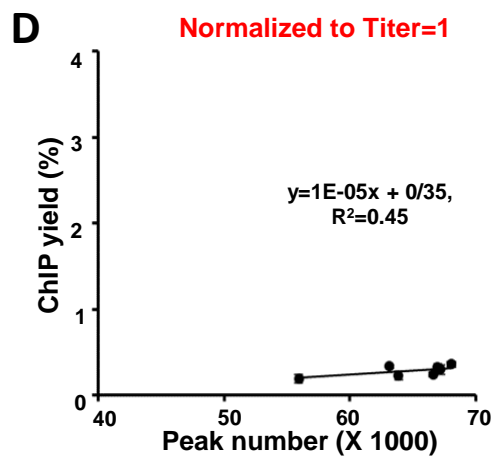

Supplement: Supplementary file 5 — Additional file 5: Figure S4. Titration-based normalization of antibody amount to the optimal titer leads to consistent ChIP yield and improved data quality. A-D: Fixed 0.25 µg of anti-H3K27ac antibody or normalized amounts to the optimal titer were immunoprecipitated with DNAchrom ranging 0.31 – 20 µg in ChIP reactions. ChIP yield was compared with the fold enrichments of H3K27ac-positive PABPC1-TSS over H3K27ac-negative MYT1-TSS loci in individual reactions of fixed antibody amount (A) and normalized amounts to the optimal titer (B). ChIP yield was compared with peak numbers from individual reactions of fixed antibody amount (C) and normalized amount to the optimal titer (D). The result was presented by the linear regression model. [file 12864_2023_9253_MOESM5_ESM.pdf]

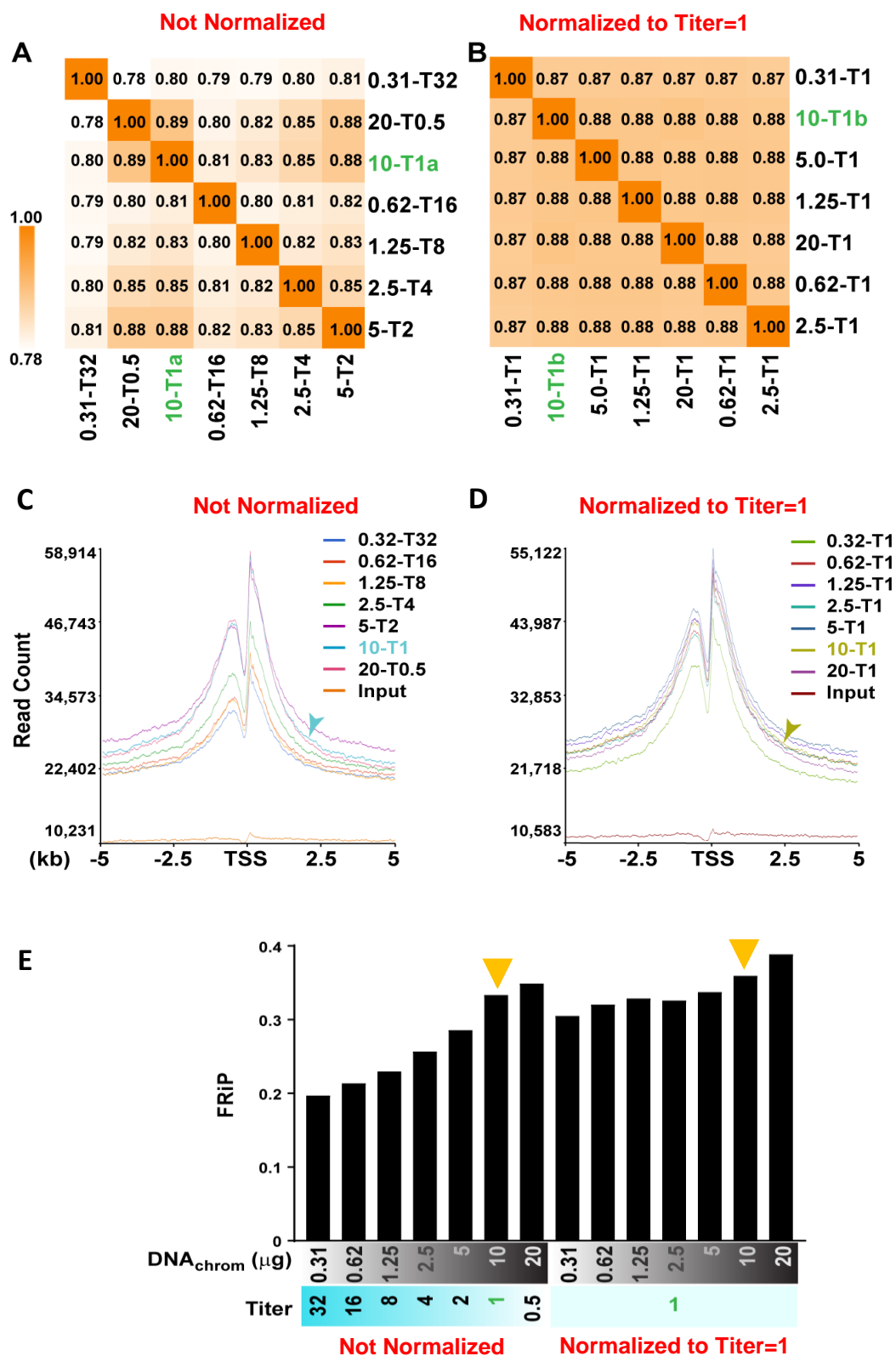

Supplement: Supplementary file 7 — Additional file 7: Figure S5. The quality and consistency of ChIP-seq libraries generated by the titration-based normalization approach. A-B: Correlation analysis between ChIP-seq datasets generated from different antibody titers. The Pearson’s correlation coefficient was visualized for the libraries generated from the conditions with the fixed amount of antibody (A) and normalized antibody amount to the optimal titer (B) in ChIP reactions. The library ID indicates DNAchrom (µg)-antibody titer in ChIP reactions. The libraries generated from 10 µg of DNAchrom was highlighted as green. C-D: Read intensities in promoter-associated peaks. Read count was visualized at peaks around TSSs for the libraries generated from fixed (C) or normalized (D) antibody amount. E: Fraction of reads in peaks (FRiP) is negatively correlated with antibody titer but improved when the antibody amount is normalized to the optimal titer. The score of FRiP was calculated by following the ENCODE guideline and visualized with DNAchrom and antibody titer. The libraries generated from 10 µg of DNAchrom was indicated as orange arrow. [file 12864_2023_9253_MOESM7_ESM.pdf]

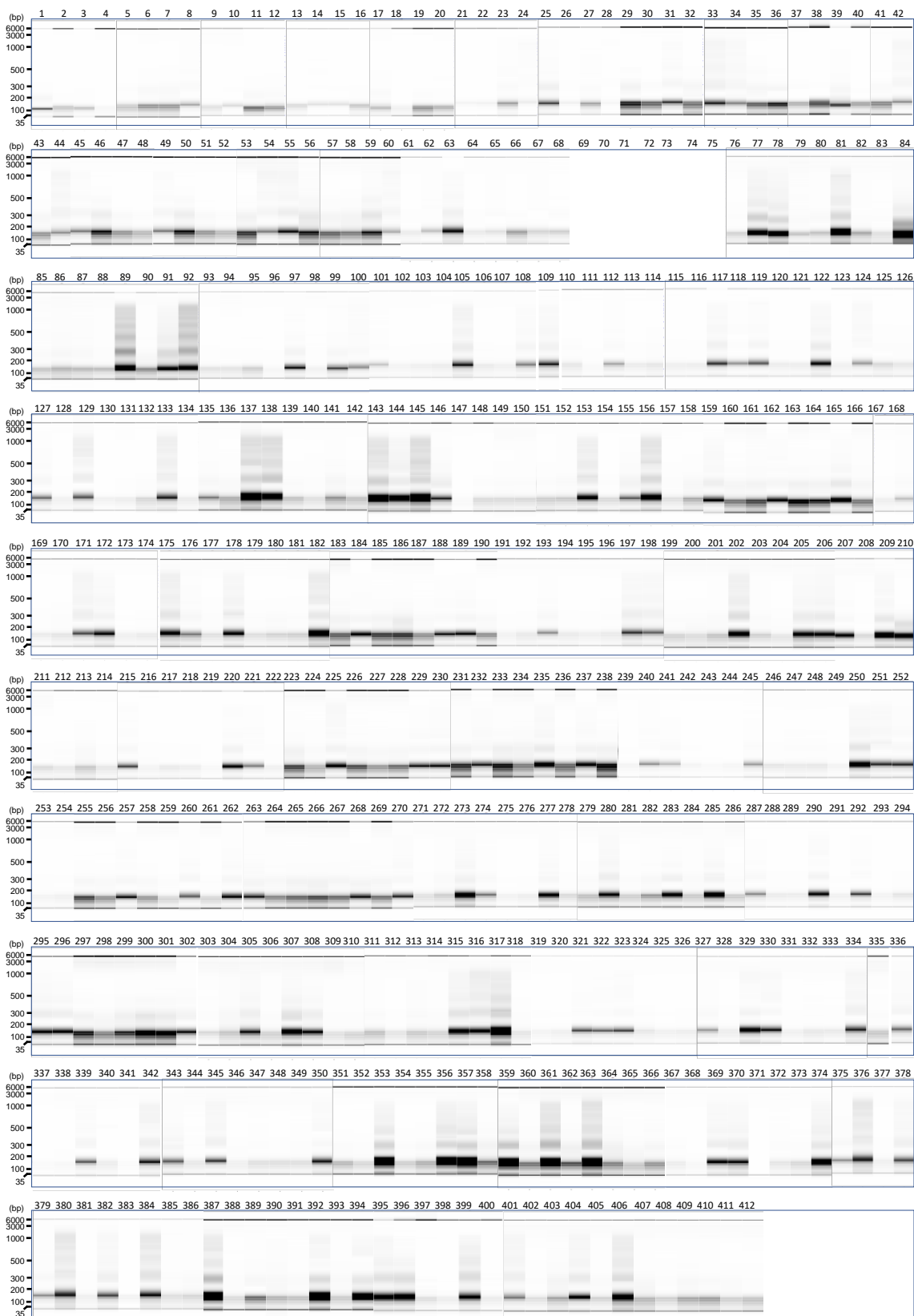

Supplement: Supplementary file 8 — Additional file 8: Figure S6. The profiles of purified DNA from chromatin input. DNA was purified from input chromatin after cross-linking reversal, RNase treatment, and proteinase K treatment. Purified DNA was analyzed by the Fragment analyzer (FA). The number indicates sample ID with the order of sample processing. Note: the FA analysis of purified input DNAs from the samples 69 - 75 was failed but we assumed the input profile is similar based on the sizes of their library DNAs. [file 12864_2023_9253_MOESM8_ESM.pdf]

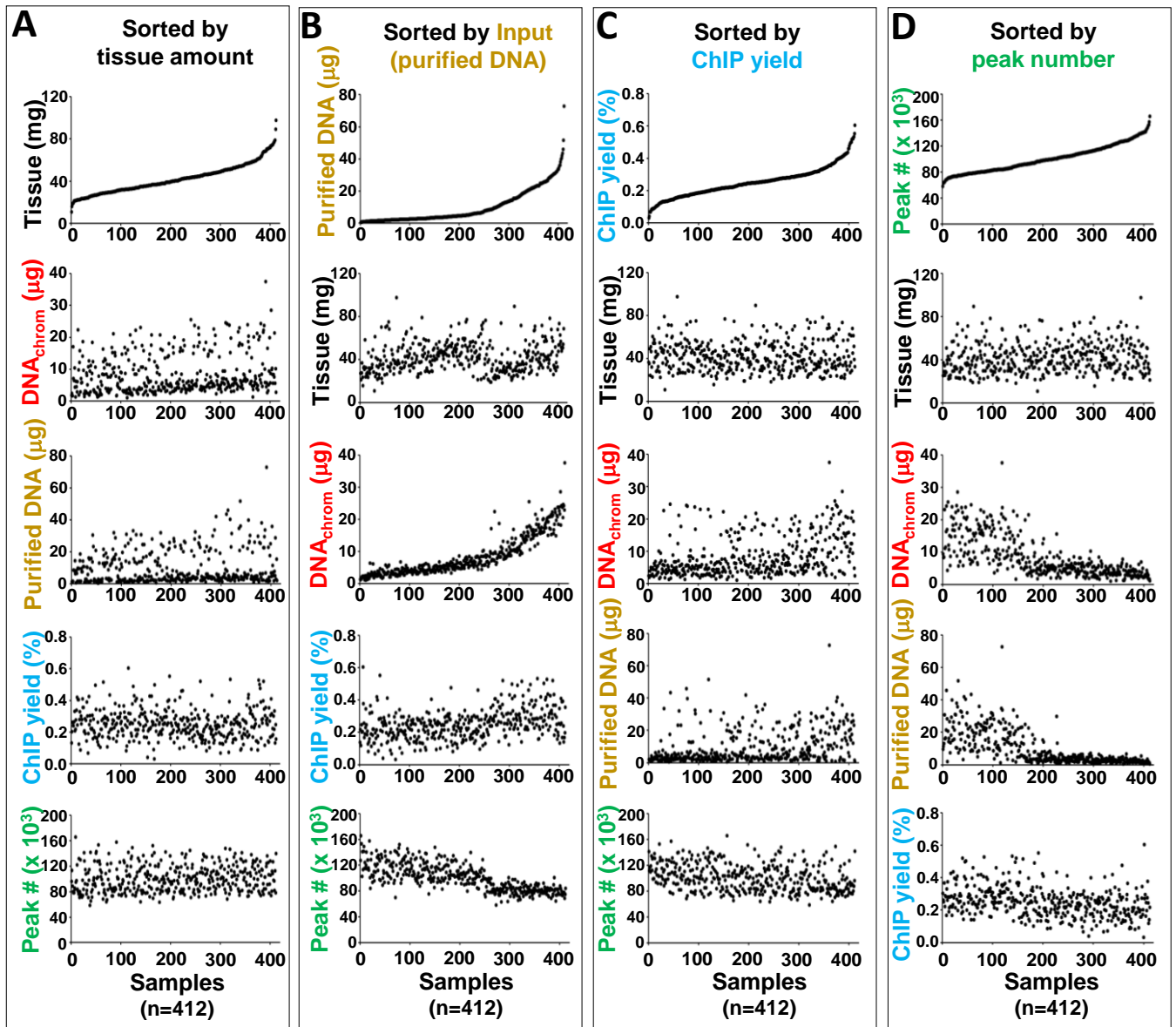

Supplement: Supplementary file 11 — Additional file 11: Figure S7. The analysis after sorting shows the consistency of experimental outcome. A-D: Collected data was sorted following tissue weight (mg) (A), chromatin input by purified DNA (µg) (B), ChIP yield (%) (C), and peak number (D), and the individual sorted data were compared with other datasets. [file 12864_2023_9253_MOESM11_ESM.pdf]

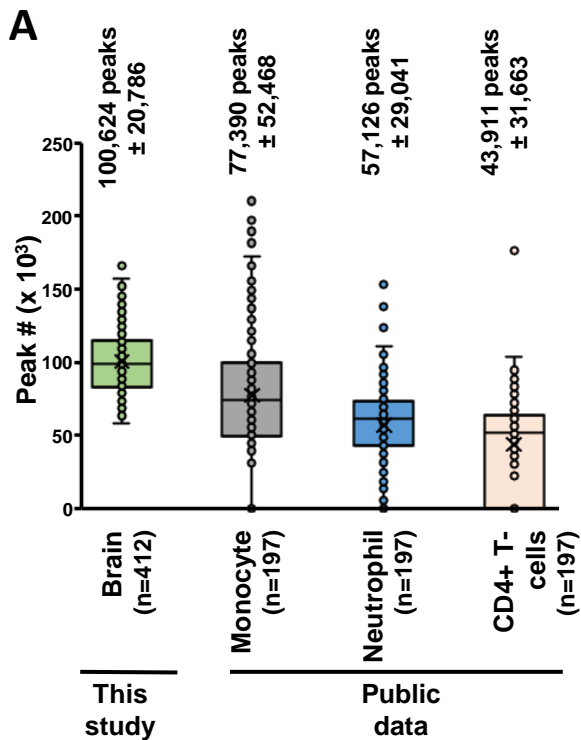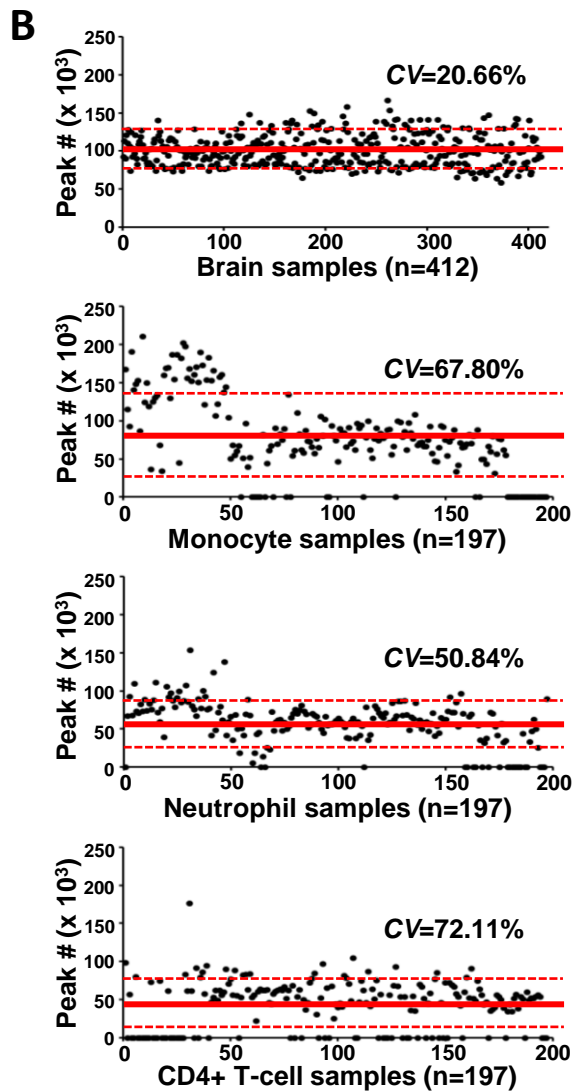

Supplement: Supplementary file 12 — Additional file 12: Figure S8. Comparison of peak number throughout the samples in large-scale H3K27ac ChIP-seq projects. A: The average peak numbers from this study and publicly available projects. The peaks were called by MACS2 using FDR<0.01. The peak number from the libraries with failed QC matrix was considered as 0. The peak number was presented as mean ± SD in box plot. B: The coefficient of variation (CV) was shown in each plot to determine the variability of results throughout the samples. The similar y-axis scales as the distribution of peak number from monocytes were used for other plots. The peak number from the libraries with failed QC matrix was considered as 0. The solid line in each plot indicates the mean value of peak number and the dashed lines indicate standard deviation (SD). [file 12864_2023_9253_MOESM12_ESM.pdf]
