# Supplementary material for: Titration-based normalization of antibody amount improves consistency of ChIP-seq experiments
Source: BMC Genomics. 2023 Apr 4;24:171. doi: 10.1186/s12864-023-09253-0 (PMC10074837; doi:10.1186/s12864-023-09253-0)
Supplement: Supplementary file 6 — Additional file 6: Table S2. Mapping results of ChIP-seq libraries used in Fig. 3. Raw sequencing reads were processed and analyzed using the ChIP-seq analysis package in the Partek Flow software. BWA aligner was used for mapping to the human reference genome (hg19). Peaks were called using the MACS2 algorithm at FDR <= 1%. [file 12864_2023_9253_MOESM6_ESM.pdf]

Library Name	Algorithm	Total # of Read Pairs	Total # Uniquely Mapped Pairs	% Unique Pairs	Complexity	Total # of Peaks
Input1		20,458,302	15,953,235	77.98	0.9557	
0.31-T32, H3K27ac	Macs	22,023,719	17,111,760	77.70	0.9606	45507
0.62-T16, H3K27ac	Macs	21,370,956	17,068,661	79.87	0.9601	51968
1.25-T8, H3K27ac	Macs	20,323,601	16,076,059	79.10	0.9611	53496
2.5-T4, H3K27ac	Macs	21,363,441	16,819,212	78.73	0.9611	60606
5-T2, H3K27ac	Macs	24,868,780	19,073,720	76.70	0.9605	65621
10-T1a, H3K27ac	Macs	21,673,372	16,715,265	77.12	0.9616	64103
20-T0.5, H3K27ac	Macs	21,480,998	16,394,830	76.32	0.9606	61087
Input2		21,751,636	16,890,922	77.65	0.9544	
0.31-T1, H3K27ac	Macs	26,805,208	15,472,204	57.72	0.9337	55909
0.62-T1, H3K27ac	Macs	24,828,411	16,592,987	66.83	0.9410	63850
1.25-T1, H3K27ac	Macs	23,089,875	16,908,899	73.23	0.9534	67151
2.5-T1, H3K27ac	Macs	20,645,152	15,862,333	76.83	0.9528	63135
5-T1, H3K27ac	Macs	21,979,962	16,909,935	76.93	0.9553	68024
10-T1b, H3K27ac	Macs	19,945,036	15,363,689	77.03	0.9565	66943
20-T1, H3K27ac	Macs	18,844,718	14,428,559	76.57	0.9545	66565

Additional file 6: Table S2
